# Can Chinese Herbal Medicine Improve Outcomes of In Vitro Fertilization? A Systematic Review and Meta-Analysis of Randomized Controlled Trials

**DOI:** 10.1371/journal.pone.0081650

**Published:** 2013-12-10

**Authors:** Huijuan Cao, Mei Han, Ernest H. Y. Ng, Xiaoke Wu, Andrew Flower, George Lewith, Jian-Ping Liu

**Affiliations:** 1 Centre for Evidence-Based Chinese Medicine, Beijing University of Chinese Medicine, Beijing, China; 2 Department of Obstetrics and Gynecology, The University of Hong Kong, Hong Kong, China; 3 Department of Gynecology, The first affiliated hospital, Heilongjiang University of Chinese Medicine, Heilongjiang Province, China; 4 School of Medicine, University of Southampton, United Kingdom; The National Institute for Health Innovation, New Zealand

## Abstract

**Background:**

A large number of infertile couples are choosing Chinese herbal medicine (CHM) as an adjuvant therapy to improve their success when undergoing *in vitro fertilization* (IVF). There is no systematic review to evaluate the impact of CHM on the IVF outcomes.

**Objective:**

To evaluate the effectiveness of CHM with concurrent IVF versus IVF alone on the outcomes of IVF and its safety.

**Methods:**

The protocol of this study is registered at PROSPERO. Eligible RCTs searched from 8 databases which compared a combination of CHM and IVF with IVF alone were included. Two authors independently selected studies, extracted data and assessed methodological quality. Meta-analysis of RCTs was conducted if there was non-significant heterogeneity (evaluated by *I^2^* test) among trials. All statistical analysis was performed using RevMan 5.1 software.

**Results:**

Twenty trials involving 1721 women were included in the meta-analysis. Three trials were evaluated as having an unclear risk of bias. The remaining trials were evaluated as having a high risk of bias. Combination of CHM and IVF significantly increases clinical pregnancy rates (OR 2.04, 95%CI 1.67 to 2.49, p<0.00001) and ongoing pregnancy rates (OR 1.91, 95%CI 1.17 to 3.10, p = 0.009). Use of CHM after embryo transfer had no better outcome in reducing the rate of ovarian hyper stimulation syndrome (OR 0.39, 95%CI 0.14 to 1.11, p = 0.08).

**Conclusions:**

This meta-analysis showed that combination of IVF and CHM used in the included trials improve IVF success, however due to the high risk of bias observed with the trials, the significant differences found with the meta-analysis are unlikely to be accurate. No conclusion could be drawn with respect to the reproductive toxicity of CHM. Further large randomized placebo controlled trials are warranted to confirm these findings before recommending women to take CHM to improve their IVF success.

## Introduction

Since the first live birth resulting from in vitro fertilization (IVF) was reported in 1978 in the UK, the use of assisted reproductive technology (ART) has doubled over the last decade [1]. In 2009, total of 146,244 ART procedures were reported to the Center for Disease Control (CDC) in United States, which resulted in 45,870 (31.4%) live births and 60,190 infants [2]. Though the number of ART cycles has increased significantly, there is only a slight improvement in the implantation and delivery rates over the years [3].

A large number of infertile couples are choosing complementary and alternative medicine (CAM) as an adjuvant therapy with the intention of improving their chances of success when they undergo IVF treatment [4], [5]. Data from a prospective cohort study in the United States shows that 17% of the couples had utilized herbal therapy for infertility [6]. Another cross-sectional study [7] found 46% of Irish patients undergoing IVF admitted regular use of CHM, with 38% of them having taken CHM in the 3-month period prior to their attendance for treatment. Chinese herbal medicine (CHM) has been used as an alternative to conventional fertility treatments and appears to be effective in improving pregnancy rates compared with western fertility drugs or IVF [8]. CHM may also increase the effectiveness of clomiphene citrate for anovulation [9], [10].

Prescription of CHM is usually based on the Chinese medicine diagnostic patterns and follows a completely different rationale to conventional treatments [11]. However, biologically plausible mechanisms of action have been suggested for CHM. According to some preclinical studies, some specific herbal products appear to improve the quality of oocytes [12], [13], to enable a reduction in the dose of gonadotrophin [14] and to increase endometrial blood flow [15], [16] and thereby increasing endometrial thickness [17]. They may promote early embryonic development, and possibly improve the success rate of IVF [18], [19].

There is no systematic review rigorously evaluating the impact of additional concurrent CHM on the outcomes of IVF. Therefore, the objective of this systematic review is to evaluate the randomized controlled trials of the effectiveness of CHM with concurrent IVF versus IVF alone on the outcomes of IVF and its safety.

## Methods

A protocol of this systematic review was registered and published at **PROSPERO,** an international prospective register of systematic review, on 17^th^ December 2012 (NO. CRD42012003479), which is available at the following website: http://www.crd.york.ac.uk/PROSPERO/display_record.asp?ID=CRD42012003479.

### Inclusion Criteria of Studies

Randomized clinical trials (RCTs) with two parallel groups that compared combinations of CHM and IVF with IVF alone were included. One of the following outcomes had to be reported: clinical pregnancy (defined as presence of at least one gestational sac with or without a fetal pole), ongoing pregnancy (defined as evidence of a gestational sac with fetal heart beats at twelve weeks), and live birth (defined as delivery of a live fetus after 20 completed weeks of gestation). There was no restriction on publication types or language.

### Search Strategy

We searched PubMed, the Cochrane CENTRAL Database, EMBASE, China National Knowledge Infrastructure (CNKI), VIP Database, Chinese Biomedical Database (CBM), and Wanfang Database with following terms as abstract terms and MeSH terms from inception to December 2012: (“in vitro fertilization” OR “fertilization in vitro” OR “intracytoplasmic sperm injection” OR “assisted reproductive techniques” OR “assisted reproductive treatment” OR “oocytes” OR “egg collection” OR “embryo transfer” OR “embryo implantation” OR “in vitro fertilization”) and (“herbal” OR “herb” OR “traditional Chinese medicine”). Clinical trials were set as a limitation for searching.

We also searched relevant ongoing trials from the US equivalent Clinical Trials register (http://www.clinicaltrials.gov). All references of the studies included and excluded were hand-searched for additional relevant reports.

### Study Selection, Data Extraction, and Methodological Quality Assessment

Two authors (CHJ and HM) independently selected studies and extracted data on patient characteristics, treatment details and outcomes. Consensus was reached by discussion with the third author (LJP) in case of discrepancy.

Two authors (CHJ and HM) independently assessed the methodological quality of the included trials. The methodological quality of RCTs was assessed according to the risk of bias tool described in the Cochrane handbook for systematic reviews of interventions [20]. Six elements were assessed: random sequence generation, allocation concealment, blinding of outcome assessors, incomplete outcome data, selective reporting and other bias. Disagreements were resolved by discussion with the third author (LJP). Due to the unequivocal outcome measurements (such as live birth, clinical pregnancy), we thought that lack of blinding of participants and personnel may not increase the risk of bias, thus we removed this item from the original 7-item risk of bias table.

### Data Analysis

All statistical analysis was performed using RevMan 5.1 (The Cochrane Collaboration) software. Pooled odds ratio (OR) with 95% confidence interval (CI) of achieving a clinical pregnancy, live birth, ongoing pregnancy and complications were measured for women receiving CHM compared with those without CHM in addition to IVF procedure. Heterogeneity of included trials was evaluated statistically using the *I^2^* test. Meta-analysis of RCTs was conducted, if there was non-significant baseline difference of participants, and if CHM intervention and control, and outcome, and the statistical (according to *I^2^*) heterogeneity test showed no significant difference among included trials. A funnel plot with ten or more studies in an analysis was used to explore the possibility of small study effects.

We conducted subgroup analyses to determine the evidence for the administration of CHM at different times during IVF (before, during and/or after the IVF). Sensitivity analyses were conducted to determine whether the conclusions would have differed if: 1) eligibility were restricted to studies without high risk of bias; 2) a fixed effect/random effect model had been adopted; 3) the summary effect measure was relative risk rather than odds ratio.

### Overall Quality of Body of Evidence: Summary of Findings (SOF) Table

A SOF table was generated using GRADEPro software (Version 3.2 for Windows). This table evaluated the overall quality of the body of evidence for clinical pregnancy, live birth, and ongoing pregnancy, using GRADE criteria (study limitations, consistency of effect, imprecision, indirectness and publication bias).

## Results

### Results of the Literature Search

**Figure 1:**
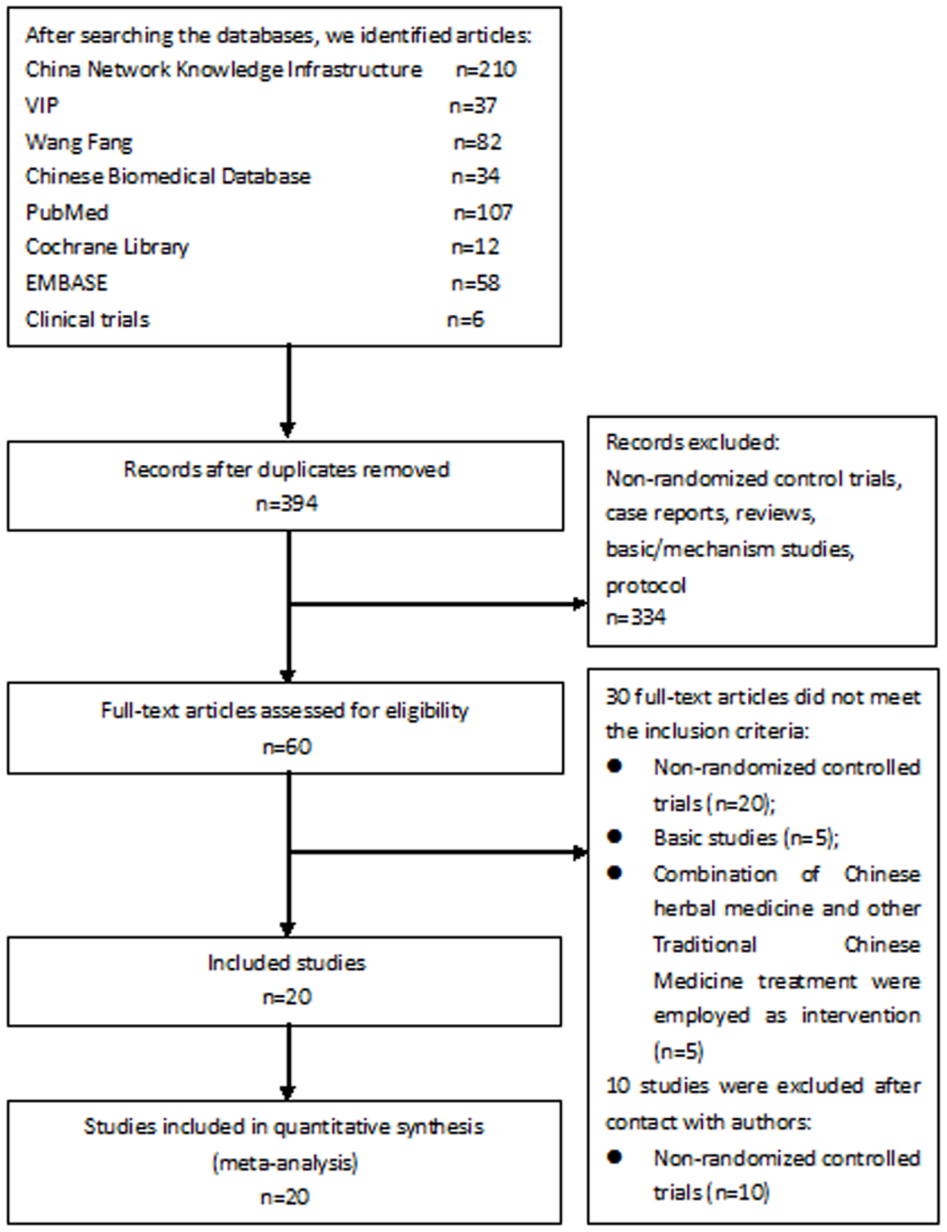
Flow diagram.

The literature search process and study inclusion are shown in Figure 1. Thirty trials met the inclusion criteria but ten studies (Table S2 in File S1) were found to be non-randomized trials after the respective authors were contacted by phone. Twenty trials [14], [16], [17], [21]–[37] were included. Two trials [31], [33] had both a published and an unpublished version: one as dissertation [38] and the other as a conference abstract [39]. Three trials were reported as dissertations [24], [34], [35]. One trial was reported as a conference abstract [28]. The remaining 14 included trials were published in scientific journals.

### Characteristics of the Included Studies

All the included trials were conducted in China and published in Chinese. In total 1721 women participated in 20 studies with average 43 women per study group. The main causes for infertility for the majority of women were tubal infertility, polycystic ovarian syndrome, and endometriosis. Four trials [16], [23], [25], [27] only included women who had failed IVF at least once before. Detailed information for the included studies is shown in Table 1.

**Table 1:** Characteristics of 20 included trials on basic information.

Study ID	Participants (original)		Primary disease for infertility	IVF	Outcome	Risk of bias
	HG	CG				
Chang et al., 2011	35	35	ITO	GnRH-a+	FR; HQER; CPR; AR; blighted ovum rate	High
Deng et al., 2011	115	134	ITO; male factors	GnRH-a+	ET; NTE; HQER; IR; CPR; OHSS	High
*Deng et al., 2011a	41	41	ITO; male factors	GnRH-a+	ET; NTE; HQER; IR; CPR; OHSS	High
Gao and Du, 2012	30	28	non organic diseases infertility	GnRH-a+	CPR; HQER; AR; OHSS; blighted ovum rate	Unclear
*Gao et al., 2012	21	21	Unclear	GnRH-a+	CPR; ET	Unclear
Ge et al., 2010	101	106	ITO; male factors	GnRH-a+	ET; HL; FR; NTE; IR; CPR; multiple birth rate; AR	High
*Huang, 2012	82	80	Unclear	Unclear	CPR; AR	High
Lei and Luo, 2011	20	21	ITO	GnRH-a+	CPR; HQER; ET; NTE; IR; HL	Unclear
Lian et al., 2006	33(36)	33(38)	ITO	GnRH-a+	HL; CPR; ET; HQER; FR	High
Lian et al., 2008	36	28	POS	GnRH-a+	HL; FR; HQER; BPR; CPR; OHSS	High
Li, 2008	31	32	ITO; male factors	GnRH-a+	ET; CPR; FR; HQER	High
*Li, 2009	40	30	Unclear	Unclear	BPR; CPR	High
Li et al., 2012	28	29	ITO	Unclear	FR; CPR	High
Liu et al., 2008	42	40	endometriosis	GnRH-a+	FR; HQER; CPR	High
Li et al., 2008	10	10	endometriosis	GnRH-a^+/−^	FR; CPR	High
Sun et al., 2011	30(31)	31(33)	fallopian tube obstruction	GnRH-a+	ET; FR; HQER; NTE; CPR	High
Teng and Lian, 2009	27 (30)	22 (30)	deficiency of the kidney qi	FET	Chinese syndrome scores; NTE; HL; ET; CPR; IR	High
Teng and Lian, 2006	42	38	ITO; deficiency of the kidney	GnRH-a+	Chinese syndrome scores; FR; HQER; BPR; CPR	High
Zhang et al., 2007	33	33	ITO	GnRH-a+	CPR; embryo score	High
Zhu et al., 2002	66(70)	69(70)	ITO; endometriosis; male factors;	GnRH-a+	FR; ET; NTE; IR; CPR	High

Women who accepted IVF and failed once (or more) before;

**IVF**: in vitro fertilization; **HG**: Herbal group; **CG**: Control group; **ITO**: infertility of tubal origin; **POS**: polycystic ovarian syndrome; **GnRH-a**: GnRH-a gonadotropin releasing hormone agonist; **GnRH-a+**: GnRH-a long programme+corpus luteum (with fresh embryo transfer); **GnRH-a^−^**: GnRH-a short programme+corpus luteum (with fresh embryo transfer); **FET**: frozen embryo transfer; **FR**: fertilization rate; **HQER**: high quality embryos rate; **CPR**: clinical pregnancy rate; **AR**: abortion rate; **NTE**: No. of transferred embryos; **IR**: implantation rate; **OHSS**: ovarian hyper stimulation syndrome; **OPR**: ongoing pregnancy rate; **ET**: endometrial thickness; **HL**: hormone levels.

One trial was on frozen embryo transfer, three trials did not report the details of IVF, and the remaining 16 trials all employed gonadotropin releasing hormone agonist (GnRH-a) on a long or short protocol with fresh embryo transfer.

All 20 trials investigated whether CHM (including decoctions and granules) could improve the clinical (or laboratory) outcomes of IVF compared to IVF alone, but the timing of the CHM in relation to IVF differed among the trials (Table 2). Intervention groups in four trials [25], [29], [30], [32] used CHM two or three months before IVF, but none of them reported that individualized prescription were used according to syndrome differentiation. Nine trials reported that CHM was given during IVF, but three of them [21], [24], [28] started using CHM on the first day of GnRH-a injection, and the other six trials [14], [31], [33]–[36] started using patent CHM (Erzhi Tiangui Granule) on the day they began follicle stimulating hormone (FSH) injections. Three trials used CHM in intervention groups after oocyte retrieval [37] or embryo transfer [16], [27]. Three trials [17], [22], [26] employed CHM during and after IVF, and the remaining one trial [23] used CHM before, during and after IVF. The composition of CHM tested in 20 included trials is showed in Table S1 in File S1. The most commonly used herbs in the different herbal formulae included *Radix rehmanniae preparata* (in 16 trials), *Radix angelicae sinensis* (in 15 trials), and *Radix paeoniae alba* (in 12 trials), which were accounted for more than half of the trials. The use of these herbs was in accordance with Chinese medicine theory, that is, to nourish *yin* and tonify kidney, regulate *qi* and activate blood (Table 2).

**Table 2:** Characteristics of herbal intervention in 20 included trials.

Study ID	Before IVF		During IVF (acceleration of ovulation)				After IVF	
			GnRHa injection	FSH injection	HCG injection	Oocyte retrieval	Embryo transfer	
Chang et al., 2011			HD of invigorating kidney for regulating menstruation. 150 ml twice daily until HCG injection					
Deng et al., 2011			HG of reinforcing liver and kidney: Cu Huang Ti (luteotrophic) Granule, one potion per day, twice daily for 7 days	HG of replenishing qi and blood: Jing Hou (post menstrua) Zengzhi (reproduce) Granule, one potion per day, twice daily until HCG injection			HG of reinforcing liver and kidney: Cu Huang Ti (luteotrophic) Granule, one potion per day, twice daily for 16 days	
Deng et al., 2011a	Periodic herbal decoction: Follicular phase: Jing Hou (post menstrua) Zeng Zhi (reproduce) Granule; luteal phase: Cu Huang Ti (luteotrophic) Granule; menstrual phase: HG of soothing the liver and regulating qi: Jing Qian (premenstrual) Granule. One potion per day, twice daily for 3 months		HG of reinforcing liver and kidney: Cu Huang Ti (luteotrophic) Granule, one potion per day, twice daily for 7 days	HG of replenishing qi and blood: Jing Hou (post menstrua) Zeng Zhi (reproduce) Granule, one potion per day, twice daily until HCG injection			HG of reinforcing liver and kidney: Cu Huang Ti (luteotrophic) Granule, one potion per day, twice daily for 7 days	
Gao and Du, 2012			HD of dispersing stagnated liver qi for relieving qi stagnation: Xiao Yao Powder, 150 ml twice daily until HCG injection					
Gao et al., 2012	Periodic herbal decoction: Follicular phase: HD of tonify qi of the kidney and nourishing yin; ovulatory phase: HG of promoting blood circulation and relieving the depressed liver; luteal phase: HD of warming kidney and activating yang. One potion per day, twice daily for 3 months							
Ge et al., 2010			HG of reinforcing liver and kidney: Cu Huang Ti (luteotrophic) Granule, one potion per day, twice daily for 7 days	HG of replenishing qi and blood: Jing Hou (post menstrua) Zengzhi (reproduce) Granule, one potion per day, twice daily until HCG injection			HG of reinforcing liver and kidney: Cu Huang Ti (luteotrophic) Granule, one potion per day, twice daily for 7 days	
Huang, 2012							HD of reinforcing liver and kidney, replenishing qi and blood: An Tai (tocolysis) Mixture 250 ml twice daily for 14 days	
Lei and Luo, 2011			HD of nourishing kidney and blood, tune up the Chong and Ren meridian: Cu Pai Luan (acceleration of ovulation) Decoction one potion until HCG injection					
Li, 2008				HD of kidney nourishing and promoting blood circulation: adjusted Erzhi Pills (or Erxian Cuyun Decoction)and Siwu Decoction, one potion per day, twice daily for 5 days			HD of warming the kidney and nourishing blood: Wenshen Yangxue Antai Decoction, one potion per day, twice daily for 5–7 days	
Li, 2009							HD of warming the kidney and nourishing blood: Wenshen Yangxue Antai Decoction, one potion per day, twice daily for 5–7 days. Adjusted the prescription and take the decoction for another 5–7 days	
Li et al., 2012		HG of promoting blood circulation for removing blood stasis, warming and activating meridian: Hua Yu Powder, 28 g, three times daily (except menstrual phase) for 2 months						
Li et al., 2008	HG of removing blood stasis and relieving internal heat or toxin: Qu Yu Jie Du Decoction 71 g once daily (except menstrual phase) for 3 months							
Lian et al., 2006				HG of tonifying kidney, nourishing blood and regulating Chong and Ren meridian: Erzhi Tiangui Granule 3 g three times daily until HCG injection				
Lian et al., 2008				HG of tonifying kidney, nourishing blood and regulating Chong and Ren meridian: Erzhi Tiangui Granule 3 g three times daily until HCG injection				
Liu et al., 2008	HG of removing blood stasis and relieving internal heat or toxin: Qu Yu Jie Du Decoction 71 g once daily (except menstrual phase) for 3 months							
Sun et al., 2011				HG of tonifying kidney, nourishing blood and regulating Chong and Ren meridian: Erzhi Tiangui Granule 3 g three times daily until HCG injection				
Teng and Lian, 2009				HG of tonifying kidney, nourishing blood and regulating Chong and Ren meridian: Erzhi Tiangui Granule 6 g three times daily until HCG injection				
Teng and Lian, 2006				HG of tonifying kidney, nourishing blood and regulating Chong and Ren meridian: Erzhi Tiangui Granule 6 g three times daily until HCG injection				
Zhang et al., 2007				HG of tonifying kidney, nourishing blood and regulating Chong and Ren meridian: Erzhi Tiangui Granule 10 g three times daily until HCG injection				
Zhu et al., 2002						HD of tonifying kidney and invigorating spleen, tonifying qi and anti abortion: Zi Shen Yu Tai Pills 6 g three times daily for 14 days		

IVF: in vitro fertilization; GnRH-a: GnRH-a gonadotropin releasing hormone agonist; FSH: follicle stimulating hormone; HCG: human chorionic gonadotrophin; HD: herbal decoction; HG: herbal granule.

Clinical pregnancy rate was reported in all 20 trials, ongoing pregnancy rate or implantation rate were reported partially. Adverse events, including miscarriage and ovarian hyper stimulation syndrome (OHSS) were recorded in one third of the included trials. Details of the results for outcome measurements in the included trials were summarized under ‘Effect of CHM on the outcomes of IVF’.

### Methodological Qualities of the Included Studies

**Figure 2:**
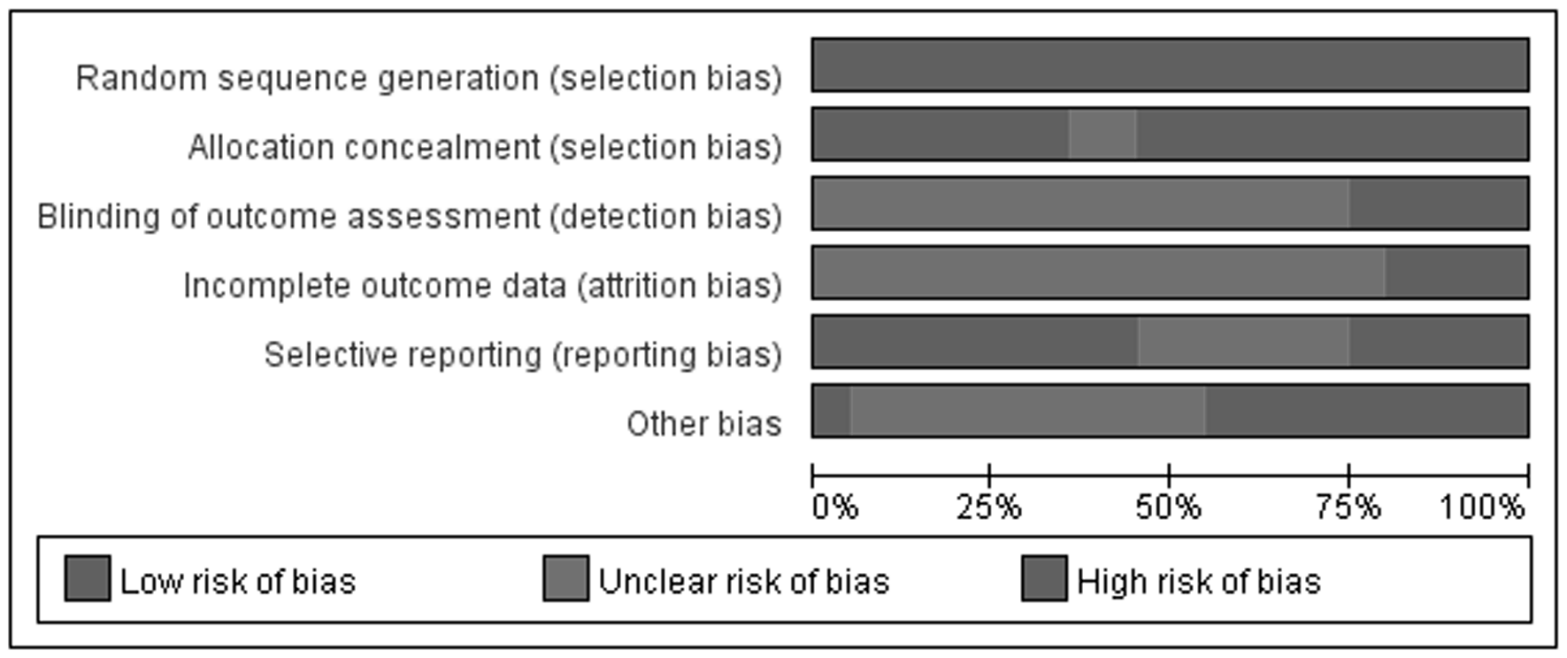
Risk of bias graph - review authors’ judgments about each risk of bias item presented as percentages across all included studies.

According to our pre-defined methodological quality criteria, three included trials [24], [25], [28] should be classified as having unclear risk of bias, and the remaining 17 trials were evaluated as having a high risk of bias (Figure 2). All trials used a random number table to generate random sequence, but only seven of them reported that allocation to specific groups was performed by a research nurse or postgraduate student. Regardless of whether blinding of participants and personnel was used (as we pre-specified), all of the trials did not provide details of blinding to outcome assessors. Four trials (Table 1) reported the number of dropouts, but intention-to-treat analysis was not employed in any study. Other biases in the majority of trials were evaluated as high risk due to insufficient information about sample size calculations, funding sources, baseline comparability and inclusion/exclusion criteria of the participants.

### Effect of CHM on the Outcomes of IVF

All the included trials compared CHM combined with IVF to IVF alone for women with infertility and they all reported the clinical pregnancy rate, which was assessed 4–5 weeks after embryo transfer. Although the component of CHM was different among the trials, the main treatment principles of the whole herbal prescription were consistent and related to the stage at which CHM was given during IVF (as showed in Table 2). All 20 trials were included in meta-analysis (Table 3) and subgroup meta-analysis was employed to address the effect of the timing of giving CHM. A random effect model was used for pooling data even though the *I^2^* test showed no significant statistical heterogeneity among trials.

**Table 3:** Estimate effect of Chinese herbal medicine for improving clinical outcomes of patients with In Vitro Fertilization (IVF).

Study ID	Total No. ofparticipants(embryoimplantation/pregnant)	Interventions	Odds Ratio [95% CI], M-H, Random model	*p* value
***1. Clinical pregnancy rate***				
1.1 herbal medicine before IVF				
Gao et al., 2012	42	Periodic herbal decoction, one potion per day, twicedaily for 3 months	2.62 [0.64, 10.61]	
Li et al., 2012	54	Hua Yu Powder 28 g, three times daily for 2 months	1.25 [0.42, 3.78]	
Li et al., 2008	20	Qu Yu Jie Du Decoction 71 g, once daily for 3 months	3.86 [0.33, 45.57]	
Liu et al., 2008	82	Qu Yu Jie Du Decoction 71 g, once daily for 3 months	2.72 [1.01, 7.32]	
**Pooling analysis 1.1 (** ***I^2^*** ** = 0%)**			**2.14 [1.14, 4.02]**	**0.02**
1.2 herbal medicine during IVF				
1.2.1 herbal medicine was given after GnRHa injection				
Chang et al., 2011	70	Fixed herbal decoction 150 ml, twice daily until HCG injection	2.65 [0.99, 7.11]	
Gao and Du, 2012	58	Xiao Yao Powder 150 ml, twice daily until HCG injection	2.33 [0.80, 6.85]	
Lei and Luo, 2011	42	Cu Pai Luan Decoction, one portion daily until HCG injection	2.57 [0.71, 9.27]	
**Pooling analysis 1.2.1 (** ***I^2^*** ** = 0%)**			**2.51 [1.34, 4.74]**	**0.002**
1.2.2 herbal medicine was given after FSH injection				
Lian et al., 2006	66	Erzhi Tiangui Granule 3 g three times daily until HCG injection	2.94 [1.03, 8.39]	
Lian et al., 2008	64	Erzhi Tiangui Granule 3 g three times daily until HCG injection	2.07 [0.67, 6.42]	
Sun et al., 2011	61	Erzhi Tiangui Granule 3 g three times daily until HCG injection	3.15 [1.10, 8.99]	
Teng and Lian, 2009	49	Erzhi Tiangui Granule 6 g three times daily until HCG injection	2.65 [0.70, 10.07]	
Teng and Lian, 2006	80	Erzhi Tiangui Granule 6 g three times daily until HCG injection	2.55 [0.99, 6.53]	
Zhang et al., 2007	66	Erzhi Tiangui Granule 10 g three times daily until HCG injection	2.74 [0.93, 8.08]	
**Pooling analysis 1.2.2 (** ***I^2^*** ** = 0%)**			**2.67 [1.72, 4.15]**	**<0.0001**
**Pooling analysis 1.2 (** ***I^2^*** ** = 0%)**			**2.62 [1.82, 3.76]**	**<0.00001**
1.3 herbal medicine after IVF				
1.3.1 herbal medicine was given immediately after oocyte retrieval				
Zhu et al., 2002	135	Zi Shen Yu Tai Pills 6 g, three times daily for 14 days	1.99 [1.00, 3.98]	0.05
1.3.2 herbal medicine was given after embryo transfer				
Huang, 2012	162	An Tai Mixture 250 ml, twice daily for 14 days	2.23 [1.19, 4.18]	
Li, 2009	70	Adjusted Wenshen Yangxue Antai Decoction, one portionfor twice daily for 10–14 days	3.33 [1.06, 10.53]	
**Pooling analysis 1.3.2 (** ***I^2^*** ** = 0%)**			**2.44 [1.41, 4.24]**	**0.001**
**Pooling analysis 1.3 (** ***I^2^*** ** = 45%)**			**2.26 [1.47, 3.47]**	**0.00502**
1.4 herbal medicine during and after IVF				
Deng et al., 2011	249	Periodic herbal decoction, one potion per day, twice daily	1.67 [1.01, 2.76]	
Ge et al., 2010	207	Periodic herbal decoction, one potion per day, twice daily	1.15 [0.67, 1.99]	
Li, 2008	63	Adjusted Erzhi Pills and Siwu Decoction during IVF, Wenshen Yangxue AntaiDecoction after IVF, one portion per day, twice daily	1.85 [0.65, 5.27]	
**Pooling analysis 1.4 (** ***I^2^*** ** = 0%)**			**1.45 [1.02, 2.06]**	**0.04**
1.5 herbal medicine before, during and after IVF				
Deng et al., 2011a	82	Periodic herbal decoction, one potion per day, twice daily	2.45 [1.01, 5.95]	0.05
**Total meta-analysis 1 (** ***I^2^*** ** = 0%)**			**2.04 [1.67, 2.49]**	**<0.00001**
***2. ongoing pregnancy rate***				
2.1 herbal medicine before IVF				
Liu et al., 2008	82	Qu Yu Jie Du Decoction 71 g, once daily for 3 months	2.04 [0.79, 5.25]	0.14
2.2 herbal medicine during IVF				
Lian et al., 2008	64	Erzhi Tiangui Granule 3 g three times daily until HCG injection	1.34 [0.48, 3.79]	
Teng and Lian, 2006	80	Erzhi Tiangui Granule 6 g three times daily until HCG injection	1.69 [0.69, 4.10]	
**Pooling analysis 2.2 (** ***I^2^*** ** = 0%)**			**1.53 [0.78, 3.01]**	**0.22**
2.3 herbal medicine after IVF				
Li, 2009	70	Adjusted Wenshen Yangxue Antai Decoction, one portion for twice daily for 10–14 days	2.97 [1.04, 8.49]	0.04
**Total meta-analysis 2 (** ***I^2^*** ** = 0%)**			**1.91[1.17, 3.10]**	**0.009**
***3. implantation rate***				
3.1 herbal medicine during IVF				
Gao and Du, 2012	58 (85)	Xiao Yao Powder 150 ml, twice daily until HCG injection	2.50 [1.03, 6.11]	
Teng and Lian, 2009	49 (120)	Erzhi Tiangui Granule 6 g three times daily until HCG injection	2.40 [0.80, 7.24]	
**Pooling analysis 3.1 (** ***I^2^*** ** = 0%)**			**2.46 [1.23, 4.93]**	**0.01**
3.2 herbal medicine after IVF				
Zhu et al., 2002	135 (492)	Zi Shen Yu Tai Pills 6 g, three times daily for 14 days	1.59 [1.02, 2.48]	
3.3 herbal medicine during and after IVF				
Deng et al., 2011	249 (489)	Periodic herbal decoction, one potion per day, twice daily	1.51 [1.04, 2.20]	
Ge et al., 2010	207 (428)	Periodic herbal decoction, one potion per day, twice daily	1.51 [1.01, 2.25]	
**Pooling analysis 3.3 (** ***I^2^*** ** = 0%)**			**1.51 [1.15, 1.99]**	**0.003**
3.4 herbal medicine before, during and after IVF				
Deng et al., 2011a	82 (225)	Periodic herbal decoction, one potion per day, twice daily	1.84 [1.03, 3.26]	0.04
Total meta-analysis 3 (*I^2^* = 0%)			1.64 [1.33, 2.01]	<0.00001
***4. adverse events***				
***4.1 abortion rate (only included trials with herbal interventions post IVF)***				
Huang, 2012	(81)	An Tai Mixture 250 ml, twice daily for 14 days	0.29 [0.09, 0.97]	0.04
***4.2 OHSS rate***				
Chang et al., 2011	70	Fixed herbal decoction, 150 ml twice daily until HCG injection	3.09 [0.12, 78.41]	
Deng et al., 2011	249	Periodic herbal decoction, one potion per day, twice daily	0.22 [0.05, 1.02]	
Gao and Du, 2012	58	Xiao Yao Powder 150 ml, twice daily until HCG injection	0.30 [0.01, 7.69]	
Lian et al., 2008	64	Erzhi Tiangui Granule 3 g three times daily until HCG injection	0.49 [0.08, 3.16]	
**Total meta-analysis 4.2 (** ***I^2^*** ** = 0%)**			**0.39 [0.14, 1.11]**	**0.08**

CI: confidence interval; GnRH-a: GnRH-a gonadotropin releasing hormone agonist; FSH: follicle stimulating hormone; HCG: human chorionic gonadotrophin.

#### i. Clinical pregnancy rate

Meta-analysis showed use of CHM significantly increased the clinical pregnancy (OR 2.04, 95%CI 1.67 to 2.49, p<0.00001, 20 trials, *I^2^* = 0%, Figure S1 in File S1). The results of subgroup meta-analysis are consistent with the overall meta-analysis.

**Figure 3:**
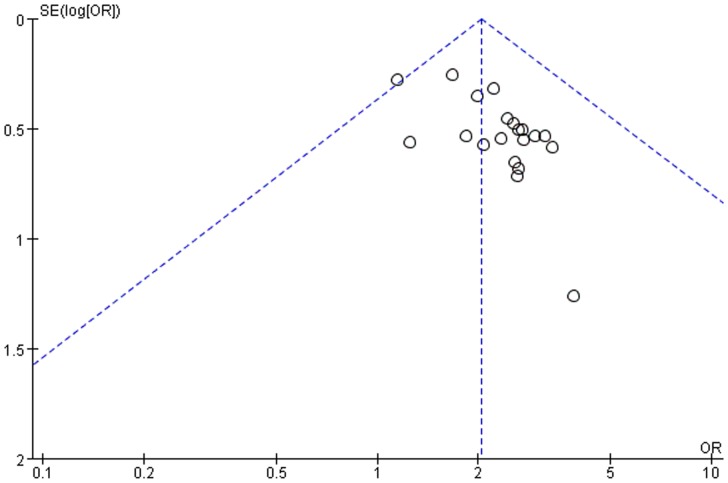
Funnel plot of Chinese herbal medicine plus IVF versus IVF alone on clinical pregnancy rate.

A funnel plot analysis of 20 trials was performed to examine outcome for the clinical pregnancy rate. The result showed obviously asymmetry (Figure 3).

#### ii. Ongoing pregnancy rate

Four trials [14], [16], [32], [35] reported the ongoing pregnancy rate. Meta-analysis showed use of CHM significantly improved ongoing pregnancies (OR 1.91, 95%CI 1.17 to 3.10, p = 0.009, 4 trials, *I^2^* = 0%). Though the results of subgroup meta-analysis showed inconsistent results of the subgroup comparing giving CHM 3 months before (OR 2.04, 95%CI 0.79 to 5.25, p = 0.14, 1 trial) or during IVF until HCG injection (OR 1.53, 95%CI 0.78 to 3.01, p = 0.22, 2 trials, *I^2^* = 0%).

#### iii. Implantation rate

The implantation rate gives the percentage of embryos which are implanted compared to the number of embryos transferred. Six trials [22], [23]–[25], [35], [37] reported implantation rates. A total of 1839 embryos were transferred to 780 women with average 2.4 embryos per woman. Meta-analysis showed use of CHM significantly increased the implantation rate (OR 1.64, 95%CI 1.33 to 2.01, p<0.00001, 6 trials, *I^2^* = 0%). The results of subgroup meta-analysis were consistent with the overall meta-analysis

#### iv. Miscarriage

Four trials [22], [24]–[26] mentioned the number of miscarriages (including blighted ovum rate). Total meta-analysis showed CHM have no significant benefit in reducing the miscarriage rate of patients undergoing IVF (OR 0.46, 95%CI 0.20 to 1.07, p = 0.07, 4 trials, *I^2^* = 0%). However, five of these eight trials which used CHM before or during IVF showed no differences between groups for miscarriage, respectively. Only one trial [27] used CHM after IVF aimed on protecting the fetus. Out of 81 pregnant women in this trial, five women in the herbal group and nine women in the control group miscarried, which showed use of CHM after embryo transfer was effective in reducing the abortion rate (OR 0.29, 95%CI 0.09 to 0.97, p = 0.04).

#### v. OHSS

Six trials [14], [21], [22], [24], [33], [37] mentioned OHSS after IVF, but only four of them [14], [21], [22], [24] reported the OHSS rate. Meta analysis showed no difference whether CHM was used before or after IVF on the number of women with OHSS (OR 0.39, 95%CI 0.14 to 1.11, p = 0.08, 4 trials, *I^2^* = 0%).

#### vi. Other adverse events

No trial followed up the patients beyond 10 gestational weeks, and the majority included trials terminated the follow up 2–5 weeks after IVF. Due to the short-term follow up duration, no trial reported adverse events relevant to reproductive toxicity of CHM [40].

#### vii. Sensitivity analysis

Sensitivity analyses were included and used to analyses whether the conclusions would have differed if the summary effect measure was RR rather than OR. The pooled results on clinical pregnancy rate (RR 1.47, 95%CI 1.32 to 1.63, p<0.00001, random model, 20 trials, *I^2^* = 0%) and ongoing pregnancy rate (RR 1.47, 95%CI 1.08 to 2.00, p = 0.01, random model, 4 trials, *I^2^* = 0%) were robust.

#### viii. Overall quality of body of evidence: summary of finding table (Table 4)

The results of 20 included trials suggest a consistent positive effect for CHM on IVF outcomes (Figure S1 in File S1). The potential clinical pregnancy rate of women with IVF who used CHM with their IVF was 53.2% (95%CI 47.7%–59.0%) compared to IVF alone (36.2%, as the median risk across studies). However, the quality of evidence for clinical pregnancy rate was “low”. This occurred because only Chinese literature was available, and there were high risks of bias (according to methodological quality assessment and funnel plot analysis) within the included studies.

**Table 4:** Summary of main findings of Chinese herbal medicine for improving clinical outcomes of patients with in

Herbal medicine compared with no other treatment for women with in vitro fertilization						
**Patient or population:** women with infertility						
**Settings:** Department of gynecology						
**Intervention:** herbal medicine combined with in vitro fertilization						
**Comparison:** in vitro fertilization						
**Outcomes**	**Illustrative comparative risks** * **(95% CI)**		**Relative** **effect** **(95% CI)**	**No of** **Participants** **(studies)**	**Quality of the** **evidence (GRADE)**	**Comments**
	**Assumed risk [control]**	**Corresponding risk [herbal medicine]**				
**clinical pregnancy** **rate** [4–5 weeks afterembryo transfer]	**[362] per 1000**	**[532] per 1000** ([477] to [590])	**OR [2.04]** ([1.68] to [2.49])	[1721] ([20])	⊕⊕⊝⊝ **low**	There were substantial and unclear or high risks with the studies as well as publication bias (according to funnel plot analysis)
**ongoing pregnancy** **rate** [2 weeks afterembryo transfer]	**[301] per 1000**	**[442] per 1000** ([325] to [602])	**OR [1.91]** ([1.17] to [3.10])	[296] ([3])	⊕⊝⊝⊝ **very low**	This data must be interpreted with great caution as the included trials are generally of poor quality and there are small numbers of trials which individually report a relatively small sample size

**CI:** Confidence interval; **OR:** Odds Ratio GRADE Working Group grades of evidence.

* The **corresponding risk** (and its 95% confidence interval) is based on the assumed risk in the comparison group and the **relative effect** of herbal medicine (and its 95% CI).

**High quality:** Further research is very unlikely to change our confidence in the estimate of effect.

**Moderate quality:** Further research is likely to have an important impact on our confidence in the estimate of effect and may change the estimate.

**Low quality:** Further research is very likely to have an important impact on our confidence in the estimate of effect and is likely to change the estimate.

**Very low quality:** We are very uncertain about the estimate.

The quality of evidence for ongoing pregnancy rate was all rated as “very low” due to the high risk of bias within the trials and the small sample sizes. Women who received combined CHM and IVF treatment had 44.2% ongoing pregnancy rate (95%CI 32.5%–60.2%), when compared to the control group (30.1% as the median risk across studies).

## Discussion

### Statement of Principal Findings

Meta-analysis of this study suggests that use of CHM (listed in Table S1 in File S1) may improve the clinical pregnancy rate (from 36.2% to 53.2%), ongoing pregnancy rate and implantation rate of the embryos for IVF, regardless of the time of concurrent administration. The clinical pregnancy rate in the control group (36.2%) and the average number of transferred embryos (2–3) are similar to data reported elsewhere [SART: www.sart.org]. However, due to the high risk of bias observed with the trials, the significant differences found with the meta-analysis are unlikely to be accurate. Thus, the findings must be interpreted with great caution as the included trials are generally of poor quality and there are small numbers of trials with a relatively small sample size.

### Strengths and Weaknesses of the Study

The methodological quality of included trials is generally poor. Most did not used method of allocation concealment and there was an inadequate method for dealing with data from women who did not reach the stage of embryo transfer after IVF. Though we evaluated the methodological quality regarding blinding to patients and personnel, none of the included trials reported the use of blinding clearly in the methods, consequently 85% of the included trials were rated as “high risk of bias”. Furthermore, due to the insufficient duration of follow-up for all of the included trials, live birth was not recorded and no trial reported the live birth rate as the main outcome measurement.

There are also limitations of the review and meta-analysis itself. Firstly, we searched the literature with general herb terms, although an abstract search was used; this may not retrieve all relevant studies, such as studies which used a specific CHM’s name but were not indexed as TCM. All the included trials were conducted and published in China and the funnel plot test showed obviously asymmetry due to the potential publication bias and small sample size of the included studies. Secondly, there was substantial clinical heterogeneity among trials with respect to the different prescriptions and usages of administration of CHM. Nine fixed prescriptions were used in 16 of the included trials. Four trials [22], [23], [25], [26] employed herbal decoctions that were adjusted according to the menstrual cycle based on fixed prescriptions (Table S1 in File S1). Though we believe that an evaluation of the whole system of CHM rather than just a particular remedy is a valid form of analysis, and the core treatment principles of the included trials were the same, complex herbal prescriptions reduced the comparability of the included studies. Even if we included all the trials in one meta-analysis regarding to the whole system of CHM for improving clinical pregnancy rate of IVF, the conclusion of this review could only be relied on the CHM which had been tested in the included trials. Thirdly, only clinical pregnancy rates and ongoing pregnancy rates were analyzed in included trials but, live birth rate should be the primary outcome for studies concerning this reproduction topic. No included trial had sufficient duration of follow up to report live births. Finally, only miscarriage and OHSS were reported in some of the included trials but no trial reported other adverse events related to CHM or congenital abnormalities of the fetuses, and because of the small sample size of the trials we could not draw any conclusions about the reproductive toxicity of CHM.

### Meaning of the Study: Possible Explanations and Implications for Clinicians and Policymakers

Although use of CHM seems to improve the clinical outcomes for women undergoing IVF, subgroup analysis highlights the impact of different outcomes related to variation in the timing of administering CHM. One trial that used CHM only for 7 to 14 days after oocyte retrieval showed less effectiveness in increasing the clinical pregnancy rate and the implantation rate of embryos (p = 0.05). This indicates that giving CHM earlier and for a longer duration may be more helpful in improving the clinical outcomes of IVF and therefore the mechanism of CHM in IVF may involve improving the quality of oocytes or embryos as suggested by some experimental studies [12], [14], [28], [34]. The results from three individual studies show CHM given before and/or during IVF may not reduce miscarriage, while application of CHM after embryo transfer was effective in reducing the miscarriage rate. The occurrence of this difference may be explained by the fact that herbal prescriptions used after embryo transfer were usually targeted at protecting the fetus [26], and involved herbals such as An Tai (Calm the fetus) Mixture, which are listed in Table S1 in File S1. Some studies also suggest that the rich content of zinc and manganese, in some medicines may have an important effect on the development of fetus [41]; other remedies may inhibit the production of potentially destructive antibodies by regulating the mother’s immunity and strengthening the immune-protective action of the mother’s immune system on the fetus [42].

### Unanswered Questions and Future Research

No published systematic review has looked at the effect of CHM in women undergoing IVF. In order to substantiate or refute these preliminary findings, it is essential that more rigorous research is carried out. True randomization, allocation concealment, blinding (if appropriate), intention-to-treat analyses and a record of live births should be routinely incorporated into research protocols. Researchers should adhere to the CONSORT standards [http://www.consort-statement.org] of trial reporting, and provide the required methodological detail. If research into CHM can incorporate these standards then its credibility will be enhanced and the potential contribution of CHM to global healthcare can be adequately evaluated.

## Supporting Information

File S1 **Figure S1. Meta analysis for clinical pregnancy rate. Table S1. Component of herbal prescriptions used in 20 included trials. Table S2. Randomized trials excluded after confirmation from contacting with trialists. Checklist S1. PRISMA checklist.**
(RAR)
